# Biological characteristics of a new human glioma cell line transformed into A2B5^+^ stem cells

**DOI:** 10.1186/s12943-015-0343-z

**Published:** 2015-04-02

**Authors:** Yanyan Li, Hangzhou Wang, Ting Sun, Jinming Chen, Lingchuan Guo, Haitao Shen, Ziwei Du, Youxin Zhou

**Affiliations:** Department of Neurosurgery and Brain and Nerve Research Laboratory, The First Affiliated Hospital of Soochow University, 188 Shizi Street, Suzhou, Jiangsu China; Department of Pathology, The First Affiliated Hospital of Soochow University, 188 Shizi Street, Suzhou, Jiangsu China

**Keywords:** Glioma stem cells, Xenograft tumor, A2B5, CD133

## Abstract

**Objective:**

The new glioma cell line SHG-139 was established and its phenotype, tumorigenicity, pathological characteristics, derived stem cells SHG139S were studied.

**Methods:**

Immunohistochemistry was used to assess expressions in the patient and mouse tumor tissues, SHG-139 and SHG-139S. Primary SHG-139 culture was performed, cell proliferation, cell cycle and genetic characteristics were assessed. MiRNA (Micro RNA) and LncRNA (Long non-coding RNA) microarray was performed.

**Results:**

We found that the glioma tissue was positive for A2B5 (Glial precursors ganglioside), GFAP (Glial fibrillary acidic protein), S-100 (Acid calcium bingding protein), VEGF (Vascular endothelial growth factor), VEGFR (Vascular endothelial growth factor receptor) and negative for Ki-67 (Nuclcar- associated antigen). SHG-139 proliferated significantly within 24h; its total number of chromosomes was 68; ratios of SHG-139 and SHG-139S cells in G1 phase were highest. SHG-139 cells were positive for A2B5, GalC (Galactocerebrosides), GFAP, S-100 and Vimentin, while SHG-139S cells were positive for A2B5, Nestin, and NG2 (Neuron-glia antigen2), and negative for Vimentin and IDHR132H (Isocitrate dehydrogenase); cells rarely stained for CD133 (Cluster of differentiation133). SHG-139 intracranial xenografts expressed GFAP, but no overt oligodendroglioma was observed. In SHG-139S xenografts, GFAP and S-100 were expressed, while CD133 was not detected; a few A2B5^+^ cells were found at tumor edges, and typical oligodendroglioma were obtained. In addition, SHG-139S xenograft tumors were more aggressive than those of SHG-139. Anti-mouse CD31 (Cluster of differentiation31) staining revealed murine vessels at the border between xenograft tumor and normal brain tissue; Anti-human CD34 (Cluster of differentiation34) staining was negative. Biochip technology of SHG139S showed several miRNA and lncRNA were differently expressed in SHG139 and SHG139S.

**Conclusions:**

SHG-139 was an astroglioma cell line which yielded stem cells SHG-139S. SHG-139S cells constituted an A2B5^+^/CD133^−^ GSC subgroup.

**Electronic supplementary material:**

The online version of this article (doi:10.1186/s12943-015-0343-z) contains supplementary material, which is available to authorized users.

## Background

Glioma is the most common intracranial malignancy in adults, and is associated with poor outcomes. Emerging evidence suggests there is a rare population of tumor cells, called glioma stem cells (GSCs), which are responsible for glioma initiation, propagation, resistance to radiotherapy and recurrence of disease [1]. We previously established the first human glioma cell line SHG-44 in china [2], which has positively impacted glioma research in our country. To further characterize glioma, this study established a glioma cell line, which can transform into glioma stem like cells with A2B5^+^. This cell line were obtained from surgical resection of brain tumor tissue of a 23-year-old male patient (database specimen No. 139, SHG-139), so this cell line was named SHG139. The patient’s clinical diagnosis was WHO II grade fibrous astrocytoma. Although there are many cell lines from GBM (Glioblastoma multiforme), permanent lines from low grade glioma have rarely been established, therefore, this cell line will fill the gap in low grade glioma biological and clinical studies. Molecular biological characteristics of the cell line, pathological and histological characteristics of intracranial and subcutaneous tumor patterns related this cell line will be detailed in this work.

## Results

### Primary culture of SHG-139 cells and their molecular characteristics

**Figure 1:**
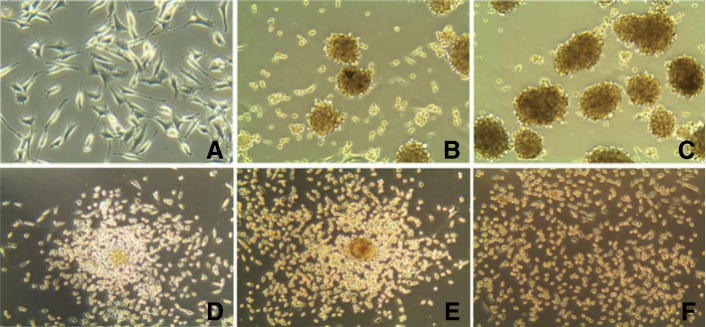
**Human glioma stem cell spheres SHG-139S derived from SHG-139 after culture in NSCM. A:** SHG-139 at the 20^th^ generation, light microscope (×400); **B, C:** SHG-139S, derived from SHG-139 at the 1^st^ and 10^th^ generations, light microscopy (×200); **D-F:** In DMEM/F12 medium without EGF, bFGF and fetal bovine serum, SHG-139s cells were adherent, cell morphology changes at 6 h, 24 h, 48 h, light microscope (×200).

**Figure 2:**
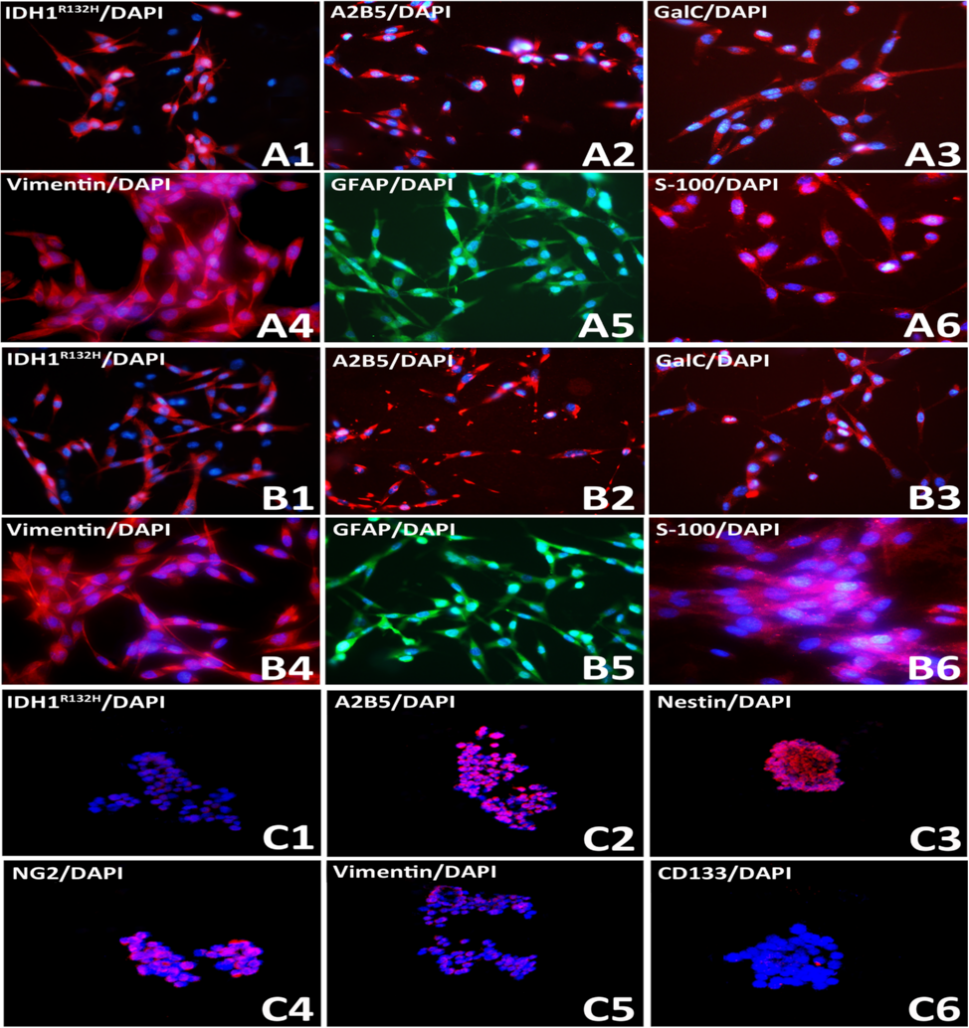
**Molecular markers expression of SHG-139, SHG139S A1-A6:.** Related markers expression of SHG139 in the 20th generation: IDH1^R132H^ (+), A2B5 (+ + +), GalC (+ +), Vimentin (+ + +), GFAP (+ + +) and S-100 (+ +), **B1-B6:** Related markers expression of SHG139 in the 60th generation: IDH1^R132H^ (+), A2B5 (+ + +), GalC (+ +), Vimentin (+ + +), GFAP (+ + +) and S-100 (+ +), **C1-C6:** Related markers expression of SHG139S in the 10^th^ generation: IDH1^R132H^ (+ +), A2B5 (+ + +), Nestin (+ + +), NG2 (+ + +), Vimentin (+ +) and CD133 (-), DAPI is a nuclear dye used as counterstain in immunofluorescence (×400).

Primary cells from fresh tumor tissues were cultured, and glioma cells grew slowly within five generations. They began to grow significantly faster at the seventh generation, and intended to be stable, cell morphology also changed. Cells were clear and mostly bipolar; somas were mostly fusiform. Single cell clone assay was carried in the 20^th^ generation of SHG-139 cells, they were adherent, with irregular cell morphology; nuclear atypia was significant, with visible round and irregular polygonal shape; cells were not clear; pseudopodia were formed in few tumor cells, while protrusion was clearly formed in most tumor cells (Figure 1, A). Immunofluorescence staining was performed with 20^th^ generation cells, and the expression of DH1^R132H^, A2B5, GalC, Vimentin, astrocyte marker GFAP and S-100 were positive (Figure 2, A1-A6).

SHG-139 cells were passaged to the 60^th^ generation, and tumor cells were adherent, and showed irregular nuclei clearly demarcated and significant cell protrusions, was similar to SHG-139 in the 20^th^ generation. Immunofluorescence staining of glioma cells (60^th^ generation), assessed by laser confocal microscopy, revealed the presence of IDH1^R132H^, A2B5, GalC, Vimentin, GFAP, and S-100 (Figure 2, B1-B6).

### Molecular characteristics of SHG-139S and its differentiated cells cultured in DMEM/F12 with fetal bovine serum

Glioma stem like cell spheres of the 10^th^ generation were assessed by immunofluorescence, 4.2 ± 1.29% SHG-139S cells in spheres were CD133^+^ (Figure 2, C1) and 84.12 ± 9.96% cells stained positive for A2B5 (Figure 2, C2). SHG-139S were confirmed as A2B5^+^ /CD133^−^ GSC (Glioma stem cells) subset. Next, the expression of different glioma molecular markers in different GSC subsets of SHG-139S were analyzed. In A2B5^+^ /CD133^−^ SHG-139S glioma stem like cell spheres, the percentages of positive cells for IDH1^R132H^ (Figure 2, C1), Nestin (Figure 2, C3), NG2 (Figure 2, C4) and Vimentin (Figure 2, C5) were respectively 36.77 ± 13.19%,73.86 ± 5.01%, 49.12 ± 12.83% and 73.37 ± 2.09%.

Cells in the 20th generation were cultured in NSCM (neural stem cell medium), and glioma stem like cell spheres were formed after 1 week in suspension, but there was still a large number of adherent cells (Figure 1, B). When passaging, only suspended cell spheres were collected and the adherent ones discarded. Until the 10th generation, a large number of glioma stem like cell spheres were observed in suspension, with only few adherent cells. These cells were named SHG-139S (Figure 1, C). SHG139S was cultured in DMEM/F12 medium without bFGF (basic fibroblast growth factor) and EGF (epidermal growth factor) and containing 10% fetal bovine serum, cells were adherent after 6h, with more adherent cells among spheres; almost all cells were adherent after two days (Figure 1, D-F).

### Cell proliferation and chromosome karyotype of SHG-139 cells

**Figure 3:**
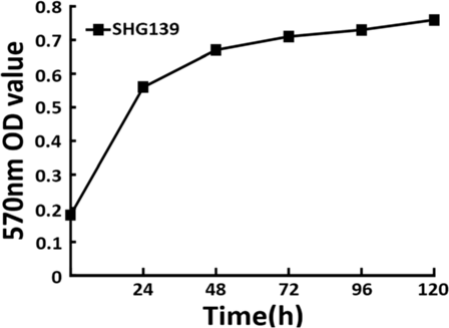
**Cell proliferation of glioma cell SHG139.** Horizontal axis is time, vertical axis is absorbance value.

Cell proliferation of SHG-139 cells was assessed by MTT assay, 570nm OD value increased obviously within 48h, was slight after 48h. The curve of proliferation showed that SHG-139 cells grew significantly within 48h, then cells grew slowly (Figure 3).

**Figure 4:**
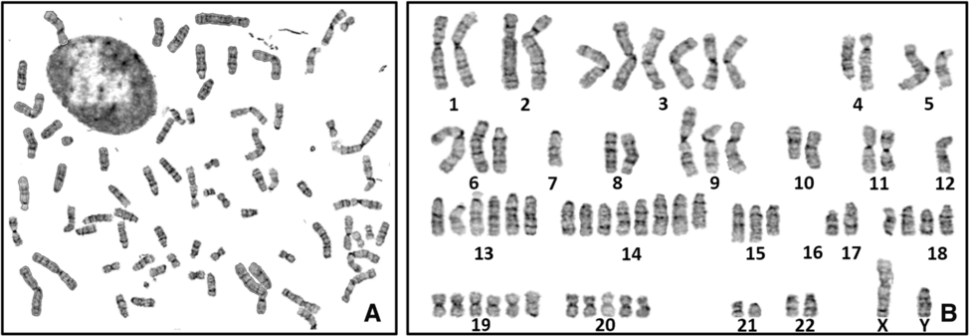
**Chromosome karyotye of glioma cell SHG139. A-B:.** Original and arrayed figure of karyotype of SHG139 cells.

SHG-139 cells of the 20^th^ generation were used for G-banding karyotype analysis after digestion, fixation, and staining. Samples were analyzed under a microscope. The total number of chromosomes was 68; their shape was irregular distortion (Figure 4, A). Chromosome karyotype pairing showed that chromosome 16 was deleted; 7, 12, X and Y were one copy; 1, 2, 4, 5, 8, 10, 11, 17, 21 and 22 were two copies; 6, 9, and 15 were three copies; 18 was four copies; 20 was five copies; 3,13, and 19 were six copies and 14 was eight copies (Figure 4, B).

### Clinical radiological and pathological data of the patient

**Figure 5:**
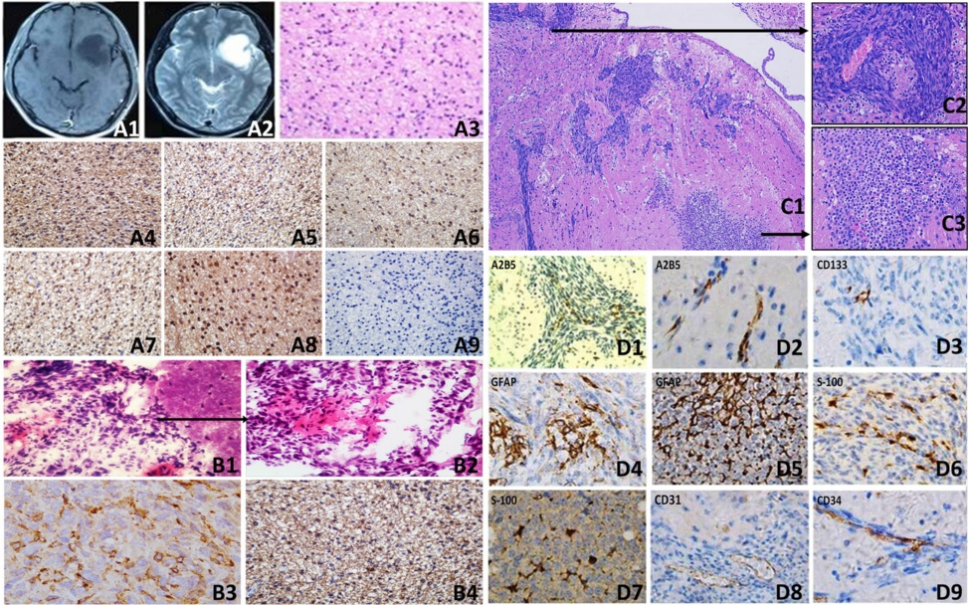
**MRI, H & E staining and immunohistochemistry data of the patient and mouse’s brain tissue. A1, A2:** Pre-surgery contrast-enhanced MRI images of T1WI and T2WI sequences of the patient; **A3:** Patient’s pathology H & E staining, light microscopy (×400); **A4-A9:** Glioma tissue immunohistochemistry: A2B5 (+ + +), GFAP (+ +), VEGFR (+ +), S-100 (+ +), VEGF (+ + +), and Ki-67 (+) (×400); **B1-B2:** H & E staing of intracranial xenograft of SHG-139, light microscope (×100, ×400); **B3-B4:** GFAP and A2B5 staining of intracranial xenograft of SHG-139, light microscope (×1000); **C1-C3:** HE of intracranial xenograft of SHG-139S, C1: Low magnification microscopy showed invasive growth of tumor cells (×100), C2: High magnification microscopy showed local area in the xenograft (×400), C3: “Fried egg-like” cells-based high cell density areas were visible in another area of the xenograft (×400); **D1-D9:** Immunohistochemistry of intracranial xenograft of SHG-139S: D1-D2: Expression of intratumor and peritumoral A2B5 (+) (×400); D3: Expression of CD133 in the xenograft (+) (×400); D4, D6: Expression of GFAP (+ +) and S-100 (+ +) in the xenograft (×400); D5, D7: Expression of GFAP (+ + +) and S-100 (+ + +) intratumor oligodendroglioma components in the xenograft (×400); D8, D9:0020cxxCD31 (+) and CD34 (+) staining (×400).

Pre-surgery MRI indicated lesions on the rear left frontal lobe, with uniform long T1, T2 signals, which were not increased significantly after enhancement (Figure 5, A1-A2). Histological examination after surgery showed overt nuclear atypia; no obvious mitotic count, necrosis and microvascular endothelial proliferation; medium tumor cell density; mostly “naked nucleus-like”; irregular nuclear hyper-chromia; loose fiber matrixes, which were formed in tumor cell protrusions; clear and visible microcapsules; WHO II grade fibrous astrocytoma (Figure 5, A3). Immunohistochemistry revealed the expression of A2B5, GFAP, S-100, VEGF and VEGFR was tan or brown dots, mainly located in the cell cytoplasm and membrane. Some S-100^+^, VEGF^+^, VEGFR^+^ nuclei were also brown. A little expression of Ki-67 was observed (Figure 5, A4-A9).

### Pathological analysis and immunohistochemistry of SHG-139 and SHG-139S subcutaneous xenografts

Subcutaneous xenografts of SHG-139 and SHG-139S were compared. subcutaneous model of SHG-139 had obvious boundary, tumor cells had a high density, with spindle-, circular- and other irregular shape and displayed overt nuclear atypia ((Figure 5, B1-B2). Immunohistochemistry of tumor was positive for GFAP and A2B5, however, obvious oligodendroglioma components were not found in this tumor model (Figure 5, B3-B4).

Intracranial SHG-139S xenografts showed invasive growth, with no obvious boundary (Figure 5, C1); tumor cells also had a high density, with spindle-, circular- and other irregular shape. Cells displayed overt nuclear atypia, with rare megakaryocytes observed and pronounced nuclear mitosis, and low degree of differentiation (Figure 5, C2). Microvascular hyperplasia was visible in some areas, with no significant vascular thrombosis and false fence necrosis changes. Oligodendroglioma ingredients featured with typical “honeycomb” and “fried egg-like” structures were identified (Figure 5, C3).

Immunohistochemical staining revealed low ratio of A2B5^+^ tumor cells, suggesting that the majority of SHG-139S glioma stem like cells expressing A2B5^+^ differentiated *in vivo* (Figure 5, D1). Meanwhile, A2B5^+^ cells were found at the edge, in a cord-like distribution, with no obvious continuity with xenograft tumor cells, indicating their aggressiveness (Figure 5, D2). A little expression of CD133 was detected in xenograft tumor (Figure 5, D3), GFAP and S-100 were detected (Figure 5, D4, D6). Interestingly, GFAP and S-100 expression was detected in oligodendroglioma ingredients of the tumor (Figure 5, D5, D7). CD31 staining showed that murine tumor blood vessels were located in the junction between the xenograft and normal brain tissue (Figure 5, D8). Indeed, xenograft tumor cells invaded outward along murine blood vessels; immunohistochemical staining with anti-human CD34 antibody yielded no signals (Figure 5, D9).

### Variant miRNA and lncRNA heatmap between SHG139 and SHG139S

**Figure 6:**
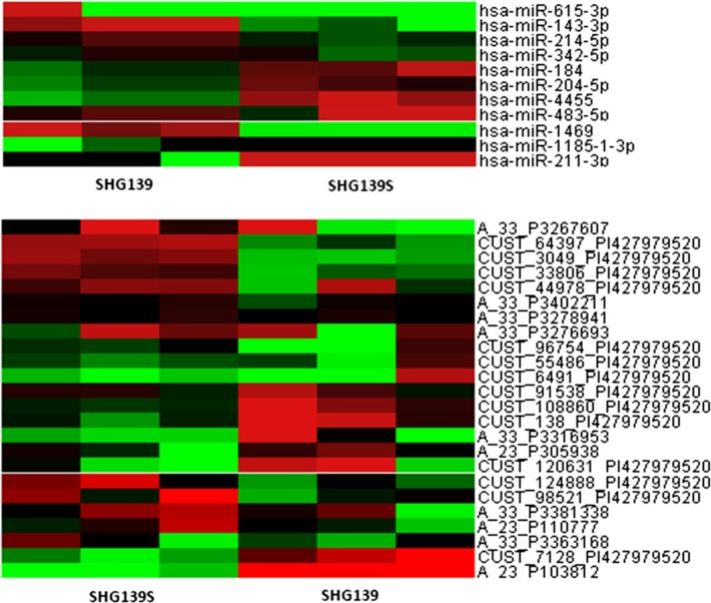
**Heatmap of variant miRNA and lncRNA in SHG139 and SHG139S.**

Total RNA extracted from SHG139 and SHG139S were treated with different methods, miRNA and lncRNA microarray analysis were performed according to relevant assays. Fortunately, we obtained variant expression of miRNA and lncRNA between SHG139 and SHG139S (Figure 6).

## Discussion

Cell culture is one of the most powerful tools in cancer research, with 60 years of history so far [3]. The earliest glioma cell lines cultured were rat glioma C6 and 9L, and human glioma U251 and U87 [4-6]. Professor Ziwei Du generated the first glioma cell line SHG-44 in our laboratory in 1984. The glioma cell line SHG-139 studied herein was gained successfully in serum-containing RPMI 1640 from WHO II grade astrocytoma (fiber type), and can be stably passaged. SHG139 in the 20^th^ and 60^th^ generations had the same molecular markers and cell morphology: GFAP, S-100 and Vimentin were expressed, and tumor cells were diploid or polyploid. Immunohistochemistry of tumor specimens showed the expression of A2B5, GFAP, S-100, VEGF and VEGFR, while Ki-67 was not detected.

Recent studies have shown that brain cancer evolves from a specific tumorigenic cell subset with highly self-renewal potential called tumor or cancer stem cells [7]. There are many culture methods for glioma stem cells: flow cytometry for molecular markers and immuno-magnetic beads are most commonly used; other methods include separation of side population (SP) cells and auto-Fluorescence [8-11]. Under NSCM with bFGF and EGF, N2 and without serum, GSCs were obtained from human glioma primary cultures or glioma cell lines, maintained parental tumor molecular phenotype, and even kept parental genotype [12-14]. This method is also known as sphere forming method; GSCs grow in the culture as suspension since they are neural stem cells (NSC) [15,16]. The new glioma cell line showed stable passage characteristics, and experimental reproducibility was good. GSCs have been gained from cell lines under NSCM in a number of studies, and the use of these GSCs has contributed significantly to reveal therapeutic targets for glioma [17-20].

In this study, SHG-139S glioma stem cell spheres were acquired from SHG-139 glioma cells under NSCM, and sphere formation rate was high. Many studies have shown that sphere formation rate of glioma stem cells is closely related with patient prognosis [21,22]. Interestingly, decreased numbers of G1 phase cells were observed in SHG-139S, while the rate of G2 phase cells was increased, compared with the values obtained for SHG-139 cells; these differences were likely related to the effects of bFGF and EGF on tumor cells. Indeed, EGF and bFGF were shown to play important roles in the development of NSC and nervous system, and self-renewal of GSCs was also closely related to EGF and bFGF [23-25].

Immunofluorescence indicated no expression of CD133, while A2B5 was detected in SHG-139s. CD133 is a well-known GSC surface marker; however, glioma stem cells with CD133^−^ have been identified [26]. Secondary sphere formation assay confirmed that SHG-139S have self-renewal capacity; immunofluorescence confirmed the expression of Vimentin in cells with the neural stem cell marker Nestin^+^; when SHG-139S were induced to differentiate in NSCM containing serum and without bFGF and EGF, adherent cells were observed, and GFAP, the early neuronal marker β-III Tubulin and the oligodendroglioma marker GalC were expressed. A2B5 was also previously confirmed as glioma stem cell marker [27,28].

With the development of GSC culture technology, GSCs with parent tumor genetics, genotype characteristics and heterogeneity characteristics in NSCM without serum, its xenograft has an advantage in affinity [29]. SHG-139S cells with A2B5 ^+^/CD133^−^were obtained in NSCM and its pathological model was established. As shown above, SHG-139 orthotopic xenograft were low invasive, but SHG-139S orthotopic xenograft model in nude mice reflected the high invasive type of GSCs. Two distinct tumor histological types in SHG139S xenograft were visible: one type was composed of irregularly shaped cells, and was similar to glial cell sarcomatoid changes; the other type showed oligodendroglioma changes and was composed of relative regular “fried egg-like” shape cells. Immunohistochemical staining of GFAP and S-100 was positive in both pathological types. The specific pathology may be associated with characteristics of GSCs (A2B5^+^/CD133^−^).

A2B5 are markers of oligodendroglioma-II astrocytes progenitors (O-2A progenitors). From the perspective of differentiation lineage, O-2A progenitor cells differentiate into oligodendroglioma or type II astrocytes cells, but it has been shown that these cells cannot differentiate into the latter, and are therefore mostly named as oligodendrocyte precursor cells (OPCs) [30]. It was proposed that oligodendroglioma tumor is formed by GSCs with A2B5 phenotype, which determines their further differentiation outcome. Meanwhile, immunohistochemistry of A2B5 in SHG-139S xenografts suggested that GSCs with A2B5+ differentiated *in vivo* and mostly lost the A2B5 molecular phenotype. Indeed, tumor cells with A2B5+ were arranged in cord-like distribution at the outer edge of the tumor, indicating their aggressiveness. In this xenograft model, anti-human CD34+ tumor cells were not found; anti-mouse CD31 staining showed murine tumor blood vessels at the edge of the xenograft, rarely in the center; anti-mouse CD34 staining revealed that murine tumor blood vessels outside the xenograft were surrounded by a high density of tumor cells, suggesting that SHG-139S cells invade outside along murine vascular system. Although SHG-139S cells with A2B5+/CD133^−^appeared with morphological changes similar to vascular mimicry in DMEM/F12 medium without EGF, bFGF and containing fetal bovine serum, this was not observed *in vivo*, suggesting that these GSCs were not closely related to formation of vascular mimicry.

Gene microarray was used to study SHG-139S and its parent cell line SHG-139, and some miRNAs and lncRNAs were different in two cells. Further experiments are underway in our group to unveil the effects of the different miRNAs and LncRNAs identified on glioma.

In conclusion, much attention should be paid on heterogeneity of glioma in order to solve current problems of chemotherapy and further targeted therapy. Comprehensive understanding of glioma cell pathology is one of the most promising ways to overcome the disease. Improvements of glioma cell culture technology, optimization of glioma model, progress of biochemical research tools, intelligence of bioinformatics, and multi-level comprehensive research tool will bring new breakthrough for basic research and clinical treatment of cancers.

## Materials and methods

### Clinical sampling and experimental animals

Glioma tissue sample was obtained from a patient at the department of neurosurgery in the First Affiliated Hospital of Soochow University in 2010. This study was approved by the ethics committee of Soochow University. The tissue in glioma sample library of our laboratory was registered as Suzhou human glioma NO: 139, so was named SHG–139. Nerve tumors’ standard of WHO in 2007 was used for diagnosis and tumor was taken out in sterile condition and packed into a sterile culture bottle and formaldehyde solution; a portion of the sample was frozen in liquid nitrogen. Twenty BALB/C nude mice were purchased from the institute of model animals of Nanjing University and housed in SPF nude mouse room of our laboratory.

### Primary cell culture and subculture of human glioma

Fresh glioma sample was washed and minced in PBS (1×) followed by 0.25% enzymatic dissociation at 37°C for 20 min. The isolated cells were resuspended in RPMI 1640 supplemented with 2 mM L-glutamine, 1% Penicilin–Streptomycin and 10% Fetal Bovine Serum (GIBCO, Invitrogen Corporation, USA). Next, cells were seeded in 25 cm^2^ culture flasks and maintained in a humidified incubator (Thermo Fisher Scientific Inc.3110, Waltham, MA, USA) with 5% CO2 at 37°C, with the culture medium replaced every other day. When cells reached 80% confluency, they were passaged after 0.25% trypsin digestion. Single cell clone was performed at the tenth generation and a stable passage cell line SHG139 was gained and authenticated successfully (Additional file 1: Table S1 and Additional file 2: Figure S1).

### Immunofluorescence assay of SHG-139 and SHG-139S

Microslides were treated with poly-L-lysine, sterile rinsed with water, UV sterilized, and dried. Single cell suspensions or sphere cell suspensions were dropped on glass slides. After fixation at room temperature with 4% paraformaldehyde, cells were blocked with goat serum and permeabilized with 0.1% Triton X-100. Primary antibodies A2B5 (mouse anti-human IgM), GC (rabbit anti-human IgG), Vimentin (mouse anti-human IgM), GFAP (mouse anti-human IgM) and S-100 (mouse anti-human IgG) were added and incubated overnight at 4°C. Cells were rinsed with PBS, and fluorescence-labeled secondary antibodies were added and incubated at room temperature, protected from light for 1h. Then cells were counterstained with DAPI for 1 min, rinsed with PBS and sealed with mounting medium. The slides were respectively analyzed by fluorescence microscopy (Olympus, Japan), confocal microscopy (Leica, German) and optical microscopy (Olympus, Japan).

### SHG-139 was transformed into glioma stem cells SHG139S and SHG139S was induced to adherent cells

SHG-139 cells of the 20^th^ generation were seeded into 6-well plates at a density of 1000 cells/well in neural stem medium composed of DMEM/F12 (Gibco, USA), 20 ng/mL EGF (Invitrogen, USA), 20 ng/mL bFGF (Invitrogen), 1% N2 (Gibco) and 1% Penicilin–Streptomycin. Cells were cultured at 37°C with 5% CO2, and cell growth was assessed under an inverted phase contrast microscope. Glioma stem cell spheres grew as suspension, and half of the culture medium was replaced for passaging. Glioma stem cells grown in suspension spheres were gently drawn when passaging, and cultured. Glioma cells derived from SHG-139 were named SHG-139S. To induce differentiation of SHG-139S spheres, they were cultured in DMEM/F12 medium contained 10% fetal bovine serum, without bFGF, EGF and other growth factors.

### Determination of glioma cell proliferation

2 × 10^3^ cells were seeded in 96-well plates and cultured in medium with 10% fetal bovine serum. A total of 6 groups were set: 0h, 24h, 48h, 72h, 96h, and 120h, each time point had three replicates. At each time point, cells in a well were treated with MTT solution (5 mg/ml in PBS) 20ul, and incubated for 4h, supernatant medium was discarded, added with 150 ul DMSO (dimethylsulfoxide), vibrated for 10 min. Cells were measured at 570 nm wavelength.

### Genetic analysis of glioma

SHG-139 cells of the 20^th^ generation were cultured for four days. After treatment with colchicine (25 μg/ml) for 6 h, preheated 0.075 mM Kcl was added for 30 min at 37°C. Then, fresh stationary liquid (methanol: glacial acetic acid =3:1) was added and cells were centrifuged at 1200 rpm for 10 min. The pellets were fixed at room temperature for 10 min (4 times), and 500 μl stationary liquid was added to resuspend the cells. Afterward, cell droplets were applied to clean microslides that had been soaked in 4 to 6°C ice water. The samples were baked in 80°C constant temperature drying oven for 15 min. After Giemsa staining, the microslides were assessed by optical microscopy.

### Pathological examination and immunohistochemistry of the patient glioma tissue

The tissue was fixed in 10% formalin, dehydrated with ethanol gradient, permeabilized with xylene and paraffin embedded. Then, serial 5 μm thick sections were cut, deparaffinized with xylene, hydrated with ethanol gradient and stained with hematoxylin and eosin (H & E) (Sigma-Aldrich, USA). Meanwhile, immunohistochemical staining was performed for A2B5, GFAP, VEGFR, S-100, VEGF, and Ki-67 detection. Endogenous peroxidase inactivation was carried out with 3% H2O2, and antigen retrieval was performed in a microwave. Afterward, primary antibodies were added to each slide at appropriate dilutions, and the sections incubated with biotin-labeled secondary antibodies for 10 min. The final signals were developed using the 3,3’-diaminobenzidine substrate (DAB) (RD, America), The sections were analyzed by optical microscopy after counterstaining with hematoxylin.

### Pathological analysis and immunohistochemistry of mouse xenograft tumor model

Glioma cells and stem cells were counted, resuspended to 1 × 10^5^ cells/μL, implanted in the right side of the brain caudate nucleus of BALB/c nude mice (n = 5). The whole process was completed in our nude SPF level room. Nude mice were anesthetized by intraperitoneal injection of 1% sodium pentobarbital, positioned on a stereotactic apparatus with prone position, and the head fixed. After alcohol disinfection of the skin of the top head, a 0.5-1.0 cm longitudinal incision was made on the frontal, 3.5 mm from cerebral midline, 2 mm frontal to coronal seam. Then the skull was carefully drilled with a dental drill. Micro-injector with10 μL tumor cell suspension was fixed on the stereotactic apparatus, and inserted vertically into brain parenchyma. The depth of the needle tip was adjusted to 3 mm from cerebral surface, and the tumor cell suspension was injected automatically for 5 min. We observed the status of nude mice with tumor after injection, including neurological deficits. Survival times were recorded and mice were sacrificed when they developed cachexia. Upon sacrifice, the whole brain tissue was removed and fixed in formalin. Pathological analysis and immunohistochemistry of A2B5_,_ CD133 (mouse anti-human IgG), GFAP, S-100, CD31 (rabbit anti-mouse IgG), CD34 (mouse anti-human IgG) of mouse xenograft tumors were performed.

### MiRNA and lncRNA microarray analysis

Total RNA was extracted using the TRIZOL Reagent (Life technologies, Carlsbad, CA, US), and assessed for amounts and integrity on an Agilent Bioanalyzer 2100. RNA samples were further purified by RNeasy mini kit (QIAGEN, GmBH, Germany) and RNase-Free DNase Set (QIAGEN, GmBH, Germany).

MiRNA were labeled by the labeling section of miRNA Complete Labeling and Hyb Kit (Agilent technologies, Santa Clara, CA, US). Each slide was hybridized with 100 ng Cy3-labeled RNA using the hybridization section of miRNA Complete Labeling and Hyb Kit (Agilent technologies, Santa Clara, CA, US) in hybridization Oven (Agilent technologies, Santa Clara, CA, US) at 55°C, 20 rpm for 20 hours. After hybridization, slides were washed in staining dishes (Thermo Shandon, Waltham, MA, US) with the Gene Expression Wash Buffer Kit (Agilent technologies, Santa Clara, CA, US). After hybridization, slides were scanned by Agilent Microarray Scanner (Agilent technologies, Santa Clara, CA, US) with default settings, Scan resolution = 5μm, PMT 100%, 5%. Data were extracted by feature Extraction software 10.7 (Agilent technologies, Santa Clara, CA, US), and normalized by Quantile algorithm, Gene Spring Software 11.0 (Agilent technologies, Santa Clara, CA, US).

Total RNA was amplified and labeled with the Low Input Quick Amp Labeling Kit, One-Color (Agilent technologies, Santa Clara, CA, US). Labeled cRNA was purified by RNeasy mini kit (QIAGEN, GmBH, Germany). Each Slide was hybridized with 1.65 μg Cy3-labeled cRNA using the Gene Expression Hybridization Kit (Agilent technologies, Santa Clara, CA, US) in Hybridization Oven. After 17 hours hybridization, slides were washed in staining dishes with the Gene Expression Wash Buffer Kit. Slides were scanned on Agilent Microarray Scanner with default settings: Dye channel, Green; Scan resolution = 3 μm, 20 bit. Data extraction and normalization was carried out as described for miRNAs.

## Additional files

Additional file 1: Table S1. Comparison of SHG139-20th, SHG139-60th and SHG139S by 9 short tandem repeat (STR) markers (DOC), 9 STR markers are same among them. Additional file 2: Figure S1. DNA Profile of SHG139 and SHG139S used in this study (DOC), the locations of STR markers were same, but peaks of each STR marker among them.
