# Supplementary material for: Biological characteristics of a new human glioma cell line transformed into A2B5+ stem cells
Source: Mol Cancer. 2015 Apr 2;14:75. doi: 10.1186/s12943-015-0343-z (PMC4392480; doi:10.1186/s12943-015-0343-z)
Supplement: Additional file 1: Table S1. — Comparison of SHG139-20th, SHG139-60th and SHG139S by 9 short tandem repeat (STR) markers (DOC), 9 STR markers are same among them. [file 12943_2015_343_MOESM1_ESM.doc]

**Additional file 1**

**Table S1. Comparison of SHG139-20th，SHG139-60th and SHG139S by 9 short**

**tandem repeat (STR) markers**

| STR marker  Cell | SHG139-20th | SHG139-60th | SHG139S |
| --- | --- | --- | --- |
| Amelogenin | X,Y | X,Y | X,Y |
| CSF1PO | 8,11 | 8,11 | 8,11 |
| D13S317 | 8,12 | 8,12 | 8,12 |
| D16S539 | 9,13 | 9,13 | 9,13 |
| D5S818 | 12,15 | 12,15 | 12,15 |
| D7S820 | 11,12 | 11,12 | 11,12 |
| TH01 | 6,9 | 6,9 | 6,9 |
| TPOX | 9,12 | 9,12 | 9,12 |
| vWA | 17, 18 | 17, 18 | 17, 18 |
