# Supplementary figures and images for: Biological characteristics of a new human glioma cell line transformed into A2B5+ stem cells
Source: Mol Cancer. 2015 Apr 2;14:75. doi: 10.1186/s12943-015-0343-z (PMC4392480; doi:10.1186/s12943-015-0343-z)

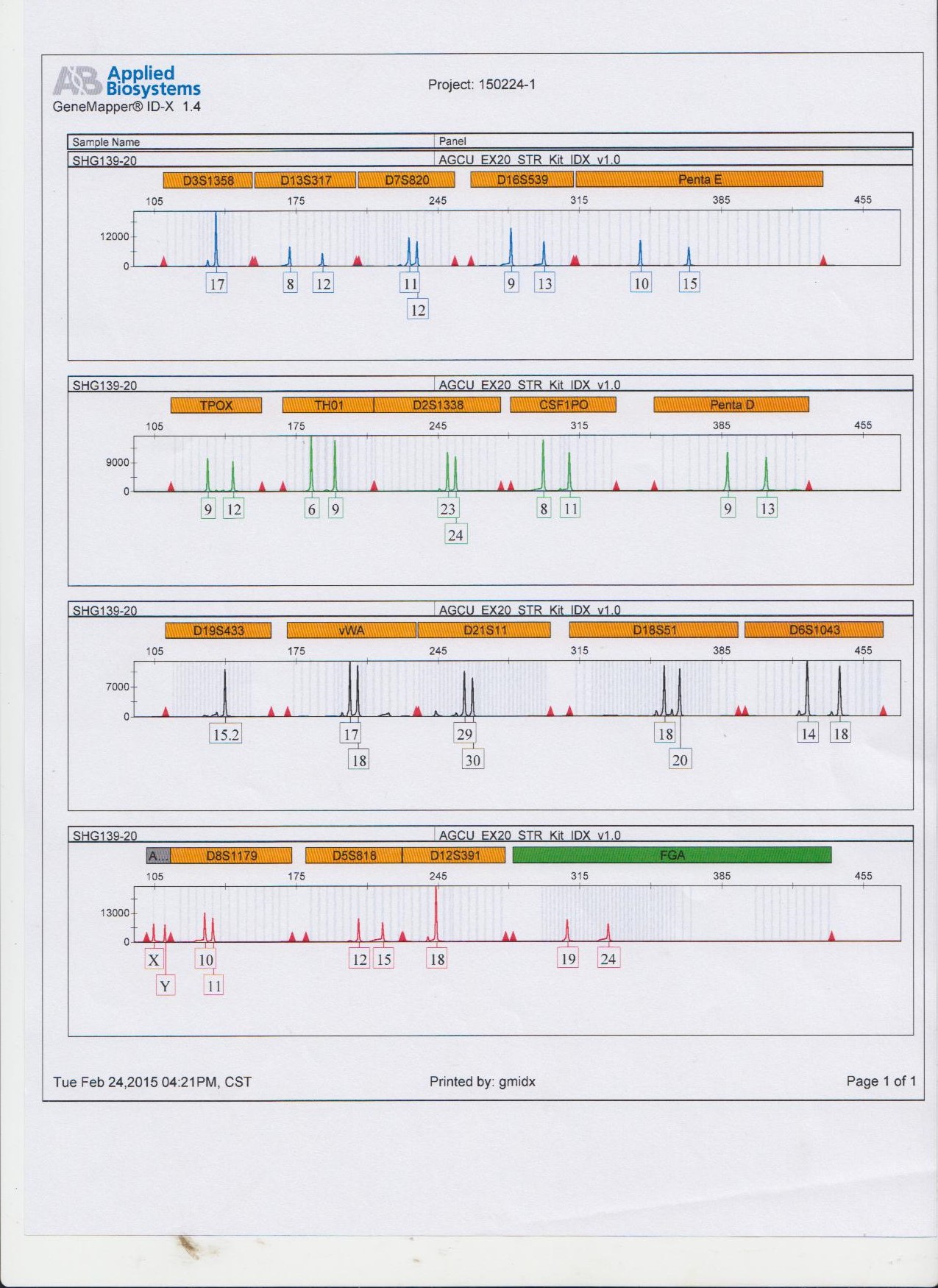


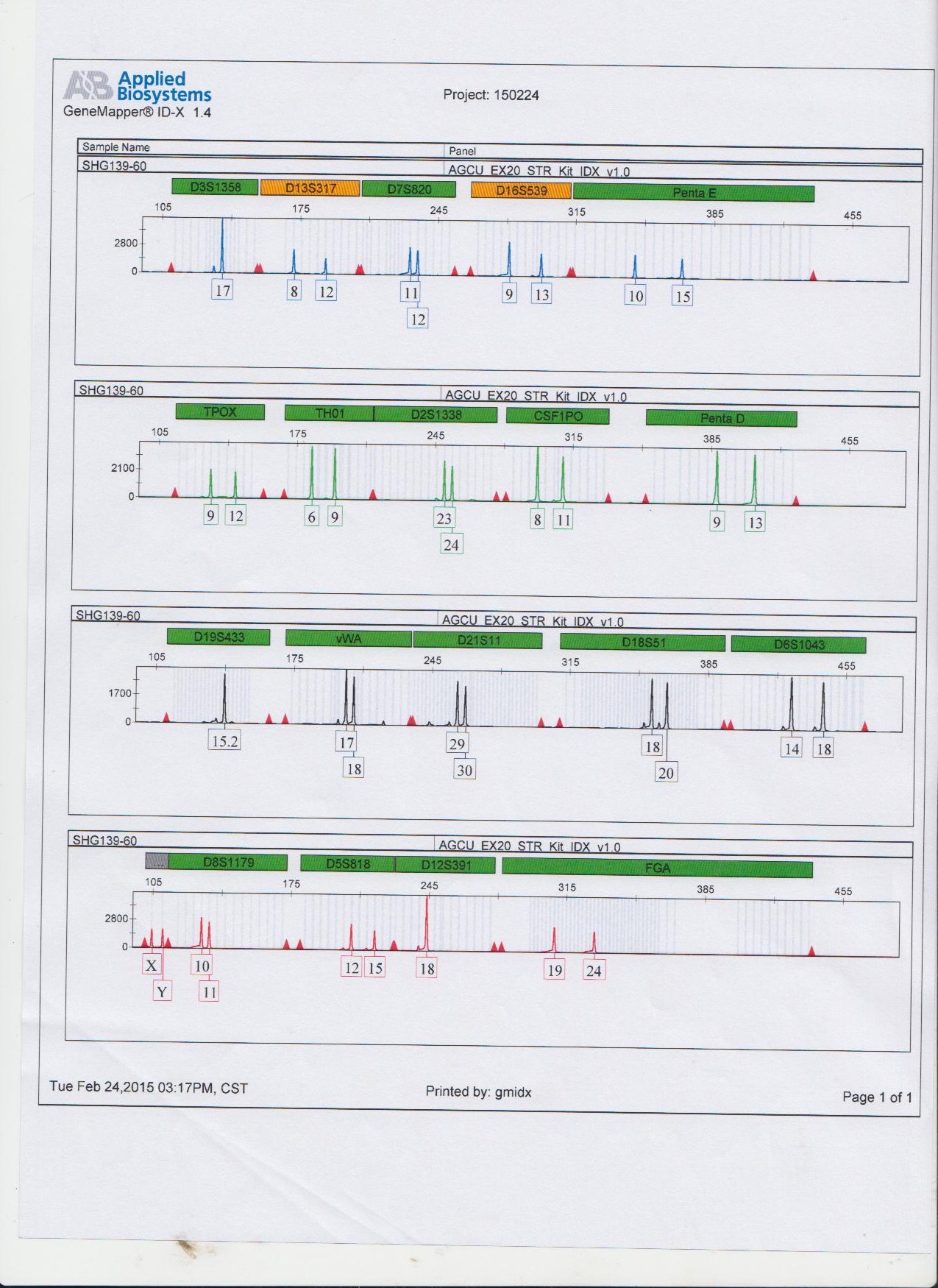


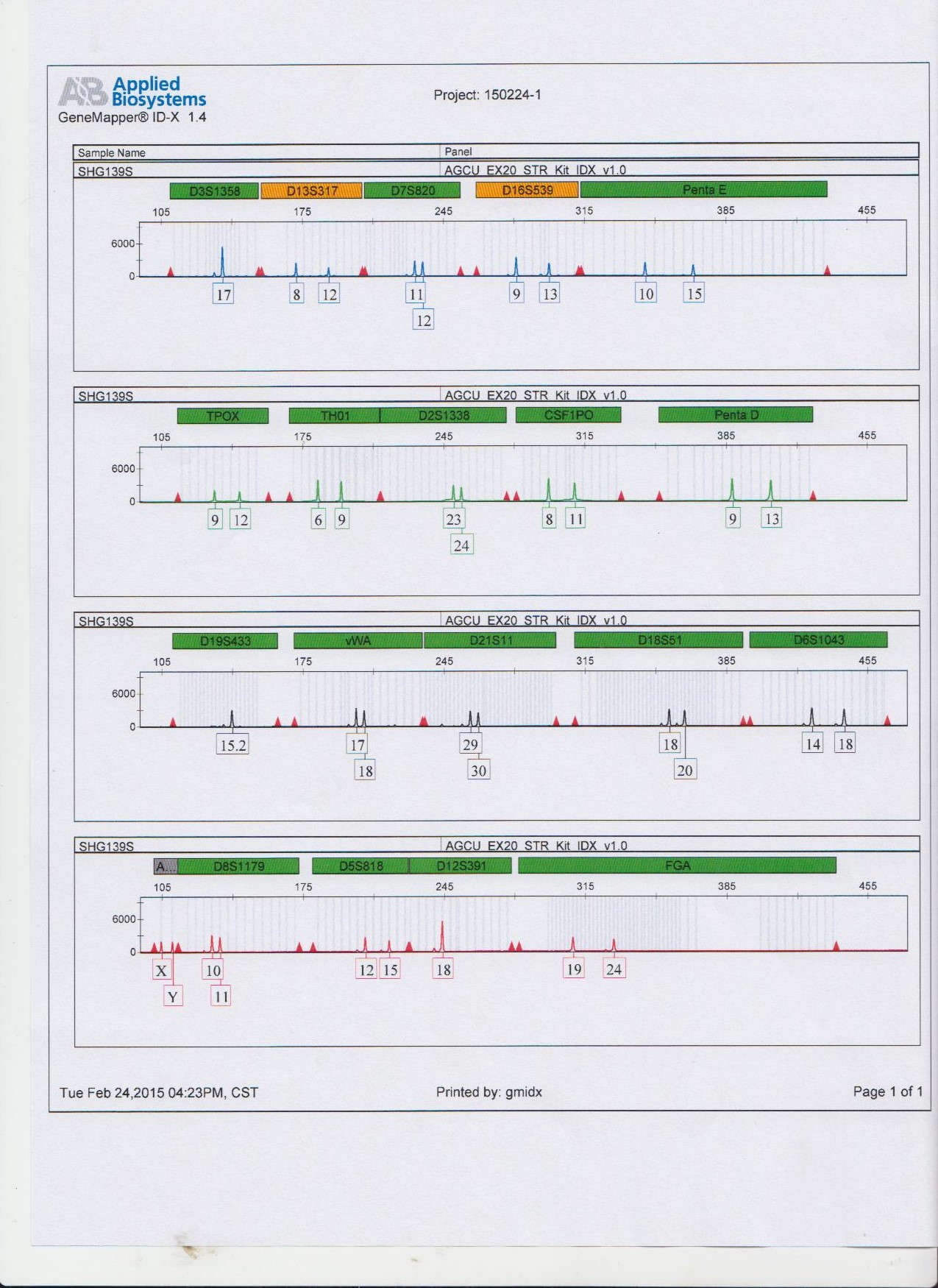

Supplement: Additional file 2: Figure S1. — DNA Profile of SHG139 and SHG139S used in this study (DOC), the locations of STR markers were same, but peaks of each STR marker among them. [file 12943_2015_343_MOESM2_ESM.doc]
